# Impoverishing effects of catastrophic health expenditures in Malawi

**DOI:** 10.1186/s12939-017-0515-0

**Published:** 2017-01-21

**Authors:** Martina Mchenga, Gowokani Chijere Chirwa, Levison S. Chiwaula

**Affiliations:** 1 Stellenbosch University, Stellenbosch, South Africa; 2 University of Malawi, Zomba, Malawi

**Keywords:** Catastrophic health expenditure, Out of pocket expenditure (OOP), Total health expenditure (THE), Impoverishment, Health financing, Malawi

## Abstract

**Background:**

Out of pocket (OOP) health spending can potentially expose households to risk of incurring large medical bills, and this may impact on their welfare. This work investigates the effect of catastrophic OOP on the incidence and depth of poverty in Malawi.

**Methods:**

The paper is based on data that was collected from 12,271 households that were interviewed during the third Malawi integrated household survey (IHS-3). The paper considered a household to have incurred a catastrophic health expenditure if the share of health expenditure in the household’s non-food expenditure was greater than a given threshold ranging between 10 and 40%.

**Results:**

As we increase the threshold from 10 to 40%, we found that OOP drives between 9.37 and 0.73% of households into catastrophic health expenditure. The extent by which households exceed a given threshold (mean overshoot) drops from 1.01% of expenditure to 0.08%, as the threshold increased. When OOP is accounted for in poverty estimation, additional 0.93% of the population is considered poor and the poverty gap rises by almost 2.54%. Our analysis suggests that people in rural areas and middle income households are at higher risk of facing catastrophic health expenditure.

**Conclusion:**

We conclude that catastrophic health expenditure increases the incidence and depth of poverty in Malawi. This calls for the introduction of social insurance system to minimize the incidence of catastrophic health expenditure especially to the rural and middle income population.

## Background

Health is considered to be a basic human right, as enshrined in the 1948, constitution of the world health organization (WHO) [1]. One of the biggest challenges to this right is cost of accessing care which may result in some financial risk by those seeking health care. The financial risks are in the form of out-of-pocket expenditure (OOP), total health expenditure (THE) as well as catastrophic health expenditure (CHE) [2–5]. CHE occurs when OOP payments for health services consume large portion of a household’s income [6]. It is estimated that 150 million people face some financial catastrophe due to OOP and that 100 million are pushed into poverty every year, as a result of CHE [7].^1^ CHE are heavily influenced by the method of financing of health care by the health sector.

Health sector financing in Malawi is composed of government financing, donor financing and private financing. Government financing is through subvention that is directed to public providers and other providers. Donor financing is through donor support to government’s development budget, commodity aid and direct support to programs and other providers. Private financing is comprised of household out of pocket expenditure, firms and insurance providers of the three [8]. While government contributions to total health expenditure have been falling, for example from 22% in 2004 to 18% in 2011, and to 13% in 2012, donor contributions have been rising from 46 to 66% of total health expenditure between 2002/03 and 2008/09 [9]. Despite the rising donor contribution, Malawi’s health system still faces absolute and relative inadequacy of financing to adequately fund its free primary health care services [10]. Consequently, about 27% of total health expenditure comes from the private sector, and from this, 53.4% is total OOP spending. This means that it is important to study OOP because it remains one of the commonest form of financing health not only in Malawi, but also the wider African continent as well [11, 12].

Malawi has no social medical insurance and an almost nonexistent private medical insurance that plays a marginal role as a source of health care finance. For instance, private health insurance managed an average of 3% of total health spending between 2007 and 2009 [13]. This is unlikely to significantly change, because Malawi has a small formal sector from which health insurance premiums could be collected with relative ease. Besides, the informal sector is characterized by low wages and salaries. Most households are therefore dependent on uninsured medical expenditures to finance health care. Financing health care through this method can pose a major threat to living standards particularly in low and middle income countries (LIMIC) with little formal health insurance coverage [14–18].

Reliance on OOP in financing health care leaves households exposed to risk of incurring large medical expenses should a household member fall sick. As such, health shocks can push households into financial catastrophe resulting from health payments and lost earnings due to inability to work [6, 15, 19–23]. It should be noted that OOP spending is incurred only when sick individuals seek care and this can increase if care seeking is delayed. In poor countries like Malawi, this burden is particularly disastrous because incomes are significantly low with almost more than half of the population living below the poverty line [24] and it is the poorer segments of the population that are highly affected.

In LIMC context, many studies have concentrated on assessing determinants of CHE and OOP and established that poverty, gender, age, education, location have significant influence [14, 16, 19, 21, 25]. Other empirical evidence also suggest that insurance offers some protection against CHE and poverty [14, 26]. It is also reported that CHE varies with the threshold (5%,10%,15%,20%, 40%) used [14, 19, 21, 27]. However only one study has tried to assess the exit time from CHE using Malawian data [28], and concluded that households have the potential if proper health insurance can be designed. Since financial protection is an ultimate goal of every health system [22, 29, 30] studying the incidence and depth of poverty due to OOP is important as it gives a picture of the extent of the financial risk and the level of protection required from the same . This creates the need to understand the effect of OOP spending on poverty, which may generate interest among policy makers to consider designing financial protection mechanisms.

The aim of the paper is to investigate effect of OOP on the incidence and depth of poverty in Malawi. The paper seeks to determine the extent of CHE due to OOP and estimate the extent of impoverishment due to OOP. Our paper adds value to literature in the sense that firstly it quantifies the incidence and depth of poverty, secondly it quantifies the extent of impoverishment due to OOP, and thirdly it quantifies the extent of poverty due to CHE, in the context where health care consumption is free at point of use. This is different to other studies cited earlier, where user fees are still used in some places and a number insurance schemes exist. Our results show that OOP pushes people into poverty even when they are accessing health for free.

## Methods

### Data sources

The data used comes from the third round of the integrated household survey (IHS3) conducted by the Malawi National Statistical Office (NSO) [31], from March 2010 to March 2011, with financial and technical support from the World Bank [24]. IHS3 was a cross sectional survey with a total sample of 12,271 households. The sample was drawn using a two-stage stratified random sampling procedure. This dataset has extensive information on household socio-economic characteristics including geographic and demographic data, and a health module. The IHS3 provides a benchmark for poverty and vulnerability indicators to foster evidence-based policy formulation, and monitor the progress of meeting the Millennium Development Goals (MDGs) (now the Sustainable Development Goals(SDGs) as well as the Malawi Growth and Development Strategy (MGDS). The survey is conducted every 5 years. The IHS3 collected data on out of pocket health spending over the past 4 weeks, and hospitalization or stay in a traditional healer’s in the last 12 months.

### Analytical methodology

The methods adopted are those proposed by Wagstaff and Doorslaer [32]. These methods have also been recommended by the World Health Organization [5] and used in a number of studies [6, 12, 14–16, 21, 25, 33–35] to investigate similar issues.

### Measuring catastrophic expenditures

In this paper, a catastrophic payment is defined based on a household’s capacity to pay [7] which measures the proportion of household income remaining after spending for basic subsistence needs [8]. Basing on this definition, indicators of catastrophic health expenditure can be measured as catastrophic head count (HC), catastrophic payment overshoot (O), and the mean positive gap (MPG). Catastrophic head count estimates the share of households in the population, whose health care costs expressed as a proportion of income exceeds a given discretionary fraction of their income *Z*. The catastrophic payment overshoot gives the average level by which payments, as a proportion of income, exceed the threshold *Z*, and the mean positive gap measures the payments in excess of the threshold average over all households.

Considering *R*_*i*_ to be the share of health care expenditure in non-food expenditure for household *i* and *Z* be the threshold beyond which household *i* incurs catastrophic expenditure, then household *i* is said to have incurred catastrophic expenditure if *R*_*i*_ > *Z*. There is no clear guidance on the value of *Z* and we decided to use four threshold (10%, 20%, 30% and 40%) based on the existing literature [5, 6, 17, 19, 23, 27]. HC is then given by:

1 $$ HC=\frac{1}{n\ }{\displaystyle {\sum}_{i=1}^n{E}_i} $$

Where *N* is the sample size and E is an indicator measure that takes the value 1 if *R*_*i*_ > *Z*. The average overshoot is defined as:

2 $$ O=\frac{1}{n}{\displaystyle {\sum}_{i=1}^n{O}_i} $$

Where *O*_*i*_ is the amount by which household $i$ share of health expenditure in non-food expenditure exceeds the chosen threshold and is estimated as:

3 $$ {O}_i={E}_i\left({R}_i - Z\right) $$

The mean positive gap (MPG) is defined as the gap over the;

4 $$ MPG = \frac{O}{HC} $$

In all the three indicators, the extent of the problem increases as the magnitude of the indicator increases.

### Measuring impoverishing effects of OOP health spending

It is very common to use household per capita consumption expenditure, as opposed to household per capita income in estimating money-metric poverty indicators. One of the disadvantages of consumption expenditure as a welfare indicate is that its measurement includes expenditures that are not welfare increasing but rather prevent the deterioration of welfare, such as some OOP health spending because spending is done when there is a sickness [7]. Poverty indicators that are based on consumption expenditure can therefore underestimate poverty levels if these expenses are not discounted. We discounted OOP health spending, from the per capita consumption expenditure, to estimate the impoverishing effects of OOP health spending.

A household is considered to be impoverished by OOP when its total per capita consumption spending falls below the poverty line (Malawi poverty line is K37002 per year^2^) after paying for health care. Therefore, the difference in the poverty headcounts before and after discounting OOP for health reflects the poverty impact of OOP or what is called the impoverishing impact of OOP.

Using the Foster–Greer–Thorbecke (FGT) indicators, *P*_*α*_ [5], poverty is measured as:

8 $$ {P}_{\alpha }=\frac{1}{n}{\displaystyle {\sum}_{i=1}^q{\left(\frac{PL-{X}_i}{PL}\right)}^{\alpha }} $$

Where *n*, is the number of households in the sample, *α* is some non-negative parameter, *PL* is the poverty line, *X* denotes per capita consumption expenditure, *i* represents individuals, and *q* is the number of households with consumption below the poverty line. The headcount (HC) index (*α* = 0), gives the share of the poor in the total population. The poverty gap (*α* = 1), is the average consumption shortfall of the population relative to the poverty line. Finally, the severity of poverty is measured by the normalized poverty gap or the squared poverty gap (*α* = 2) [5]. Poverty indicators that have netted out OOP health spending are derived as *P*_*α*_^*net*^:

9 $$ {P}_{\alpha}^{net}=\frac{1}{n}{\displaystyle {\sum}_{i=1}^q{\left(\frac{PL-{X}_i-OO{P}_i}{PL}\right)}^{\alpha }} $$

Where OOP is the per capita out of pocket health spending. The impoverishing effects *P*_*α*_^*effect*^ of OOP on poverty is then derived as differences in the poverty measures;

10 $$ {P}_{\alpha}^{effect}={P}_{\alpha }-{P}_{\alpha}^{net} $$

## Results

### Demographic and socioeconomic profile

This section provides the descriptive statistics of the households in our sample (see Table 1). About 84% of the households that were surveyed live in rural areas, and this is similar to the population as indicated by the last national Population and Housing Census (NSO 2008). The average household size is four members which is slightly lower than 4.6 reported in the most recent Population and Housing Census (NSO 2008). Slightly over half of the households, have children less than five years old, while nearly 20% of households have at least one year aged member. Nearly 70% of the households have members with at least primary education, and only 4.87% of the households have members with tertiary education. The analysis also shows that majority (76%) of households are headed by males compared to 72% reported in the 2008 census data. Having a male headed household would reduce the chances of being poor [36] and influence resource allocation decision. Finally, on average the nearest health facility was reported to be 8.59KM away, this makes accessing health care costly as they have to pay money for transport. Largely, the demographic characteristics from the IHS3 are similar with the characteristics from the census data which makes the IHS3 representative of the national population.

**Table 1:** Descriptive statistics (*n* = 12271)

Variable	IHS3 (%)
Residence	
Rural	84.40
Urban	15.60
Household size	
1	7.06
2	10.13
3	16.87
4	17.72
5+	48.22
Household age composition	
Children <5 years	59.16
Adults > 60 years	19.18
Schooling years per household	
No education	0.01
Primary	60.95
Secondary	34.26
Tertiary	4.87
Household Headship	
Male	76.01
Female	23.99
Employment status	
Employed	21.83
Unemployed	78.17
Access variable	
Distance to nearest facility	8.59KM

Source: Author based on NSO (2011) [24] data

### Annual consumption expenditure

The data indicate that the average per capita annual consumption expenditure was MK59699.80 (US$136.93), of which almost 60% was spent on food. There are significant differences in the mean values of per capita consumption expenditure across quintiles (see Table 2). The average household expenditure of the richest quintile is nearly nine times of the poorest quintile. The average expenditure of the middle quintile is almost 32% higher than the second quintile whereas the average expenditure of the fourth quintile is 34% higher than the middle quintile.

**Table 2:** Expenditure on Health and Access to Health

Quintiles	Percent	Average annual per capita consumption expenditure (MK)
Poorest	17.58	17,160.34
Poor	22.17	29,140.16
Middle	20.97	42,833.15
Wealth	21.57	64,973.40
Wealthiest	17.171	153,726.58

Source: Author based on NSO (2011) [24] data

### Catastrophic health expenditures

Table 3 presents the incidence and intensity of catastrophic health expenditures in Malawi.

**Table 3:** Incidence and Intensity of Catastrophic Health Payments in Malawi

Catastrophic payments measures	Threshold budget share, z			
OOP health spending as share of non-food expenditure	10%	20%	30%	40%
Head Count (H)	9.37%	3.41%	1.61%	0.73%
Standard Error	0.26%	0.16%	0.11%	0.08%
Overshoot (O)	1.01%	0.43%	0.20%	0.08%
Standard Error	0.04%	0.03%	0.02%	0.01%
Mean Positive Gap (MPG)	10.76%	12.64%	12.15%	11.63%

Source: Author based on NSO (2011) [24] data

The results suggest that OOP drives between 0.73 and 9.37% of the households in Malawi to encounter CHE, as we decrease the threshold from 40 to 10%. If we increase the threshold from 10 to 40%, the mean overshoot drops from 1.01% of expenditure to only 0.08%. Unlike the head count and the overshoot, the mean overshoot among those exceeding the threshold (MPO) need not decline as the threshold is raised. Those spending more than 10% of non-food expenditure, on average spent 20.76% on health care whereas those spending more than 40% of the non-food expenditure, on average spent 51.63% on health care.

### Health expenditures and poverty

Under this section we look at the effect of health care payments on the incidence and depth of poverty. We compare the headcount and poverty gap measures before and after expenditures on healthcare are taken into consideration. Table 4 demonstrates the sensitivity of poverty measures in Malawi to the treatment of health payments.

**Table 4:** Measures of Poverty Before and After Netting Out Spending on Health Care, Malawi 2011

	Gross of health	Net of health	Difference	
	Payments (1)	Payments (2)	Absolute	Relative
			(3) = (2)-(1)	[(3)/(1)]*100
Poverty Head count	50.98%	51.91%	0.93%	1.82%
Poverty Gap	0.19	0.20	0.48	2.54%
Normalized Poverty gap	9.37	9.67	0.30	3.22%

Source: Author based on NSO (2011) [24] data

The conventional methodology of measuring poverty suggests that about 50.98% of the population is poor. If OOP payments for health care are netted out of household consumption, this percentage rises to 51.91%. So about 0.93% of the Malawian population is not counted as living in poverty but would be considered poor if spending on health care is discounted from household resources. This represents a substantial rise of 1.82% in the estimate of poverty. The estimate of the poverty gap of the poor also rises by almost 2.54%, from 19% less than the poverty line to 20%. Expressed as a percentage of the poverty line, the poverty gap increases from 9.37 to 9.67% when health payments are netted out of household consumption.

### Impoverishment by location

**Fig. 1:**
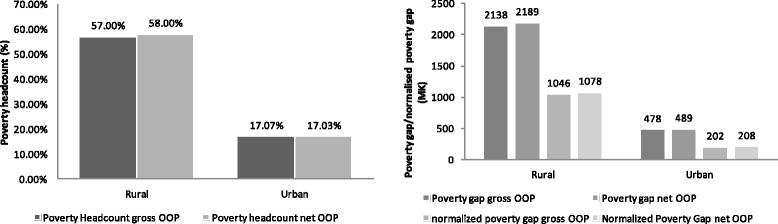
Impoverishment by Location. Constructed by author from the IHS3 dataset

Figure 1, compares the poverty impact of catastrophic health expenditures between rural and urban residents. From the figure, it can be seen that the proportion of impoverishment in the rural area is relatively larger than in the urban area. This difference can be attributed to a lot of factors, one of which is access to health facility and also that most people in the rural areas rely on agriculture and majority of them are poor and cannot afford private insurance. As such whatever little money they spend in trying to access healthcare leaves them worse off than before.

### Impoverishment by expenditure quintile

**Fig. 2:**
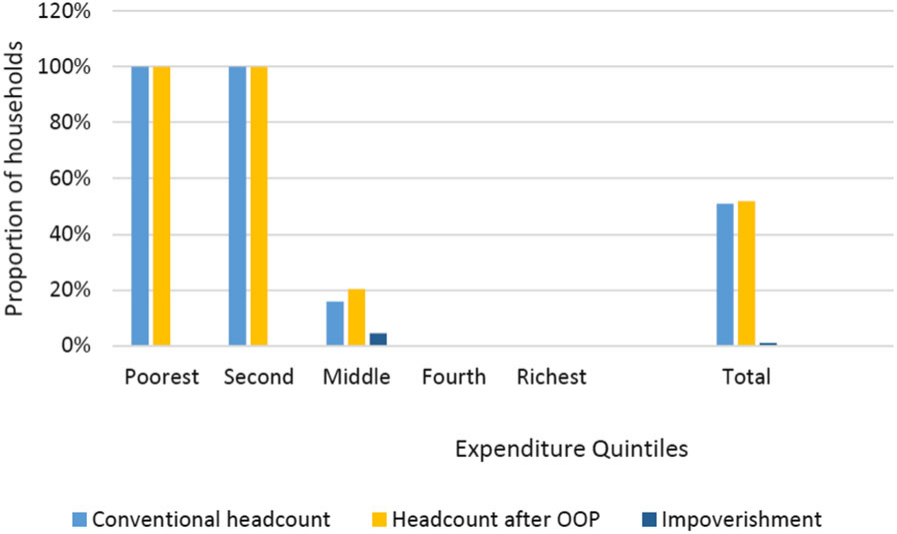
Impoverishment by economic status. Constructed by author from IHS3 dataset

Figure 2, compares the poverty impact of catastrophic health expenditures across income quintiles. It can be seen that the proportion of impoverishment at poorest quintile is negligible, as households in the poorest quintile already live below the poverty line. However, the impact of health payments on household welfare reaches to the middle quintile, which has the highest proportion of households being pushed into poverty due to health care payments. Negligible amount of the households at fourth and richest quintiles are impoverished by health payments.

## Discussion

We have determined the extent of catastrophic health expenditure and its impoverishing effects in Malawi where care is free at point of use in public hospitals and where no social insurance scheme exist. This is an area of policy interest given the ongoing implementation of the essential health care package and the intention to introduce user fees in some public health facilities such as central hospitals (tertiary care). Our study results have shown that OOP drives a substantial proportion of households in Malawi to encounter financial catastrophe, and that up to 0.93% of households fall below the poverty line after accounting for health care expenditure. Although the estimated incidence of catastrophic OOP expenditure may appear to be very low, the effects of the same on the households that are affected may not be undermined. These estimates also fall within the ranges that have been found by other studies [14, 15, 19, 25, 27, 33].

The results show that most of the impoverishing impact of out of pocket health expenditures is felt by people who mostly resides in the rural areas and middle income households. Not captured in this study is the effect of inability of the poor to spend on health. The inability of the poor to pay for health care limits access and this may prolong a sickness which may limit their ability to work reduce their net income. While our analyses are not without their limitations, there is no doubt that health expenditure contributes substantially to the impoverishment of households in Malawi, increasing the incidence of poverty and pushing poor households into deeper poverty. These results are consistent with earlier studies, in which poor households were less able to cope with any given level of health expenditure than richer households [11].

Both the incidence and intensity of poverty, are higher at lower thresholds, and in all cases, as thresholds increase, the mean positive poverty overshoot increases. Much of the increase in the MPO is due to a modest decline in the mean gap, relative to the headcount as the threshold is raised. This means that the ‘catastrophic’ effect of health costs was mostly felt through increase in poverty incidence than in deepening of poverty among those who are already poor. This may suggest that there is a great proportion of households that are marginally non poor.

The outcome from these results are similar to Mussa [9], who establishes that, the incidence of catastrophic payments, and the mean overshoot, which captures the intensity or gap of the occurrence of households incurring catastrophic expenditure, declines as the threshold rises. However, increasing threshold from 5 to 15% of total expenditure the incidence of catastrophic payments declines from 6.9 to 1.1%. In other words, for a given threshold, the incidence and intensity of catastrophic payments are higher when the share of ability to pay is used; and the headcounts range from about 11.3 to about 1%, and the MPOs range from 11.2 to 12.9%.

More to this, the results confirm those from Vietnam and entire Asia [7]. Van Doorslaer and others found that poverty estimates after paying for health care were much higher than conventional estimates, ranging from an additional 1.2% in Vietnam to 3.8% in Bangladesh [11]. They concluded that OOP health expenditures are likely to inflate the extent of poverty and we find it imperative to agree with their conclusion for an African setting.

## Conclusions

These findings are found on the background that public health services in Malawi are mainly free, which means that the health care expenses recorded are mainly in private health care, as well as purchase of drugs at pharmacies and grocery shops. This may be the reason why the effect of OOP on poverty gap is not large because most of the poor access health care at public facilities. The present plans by the government to introduce user fees at some major public health facilities imply that OOP will increase and this will have huge welfare effects on the population. As such we suggest that the government should consider introducing the social insurance system or government supported community based health insurance(CBHI) like the case in Rwanda, to minimize the incidence of catastrophic health expenditure, as well as reduce its effects especially in the rural areas.

This negative influence of health systems on households that can lead to impoverishment has long been ignored on the health policy agenda in Malawi. Once the issue has been identified, catastrophic payments can quickly become priorities in national health-policy debates, as is the case in Mexico and Iran. Based on the above findings, board policy areas that aim to protect households from catastrophic health payments and impoverishment should be explored. The systematic review by Ekman [12], found strong evidence that CBHI schemes “do provide effective protection to the members of the schemes by significantly reducing the level of OOP payment for care”, even though some of the evidence was mixed. There was also moderately strong evidence to suggest that CBHI schemes provide financial protection by increasing access to health care in their operating areas. Access to care was assessed mainly by measuring utilization rates, comparing members and non-members, and conducting before-after appraisals of utilization of services. Given the above limitations to the study, further research would be the next most reasonable thing to do, however, no further research has been carried to support or refute the above results.

## Limitations of the study

The interpretation of these findings needs to be tempered by the limitations of the methodologies employed. First, since health expenditure can only be incurred if sick individuals actually seek care, and those towards the lower end of the income distribution tend to face greater physical and financial obstacles to seeking care, we expect that, in general, the estimates generated by this analysis will underestimate the true effect of health expenditure on poverty, creating the impression of a greater degree of financial protection than the system actually provides.

Second, we are only measuring financial protection in the current period. OOP payments in the current period may be financed by sources other than current income, such as dis-saving, borrowing and depletion of assets, which allows households to smooth non-health consumption in the period in which ill health occurs. While this coping mechanism may protect households from impoverishment in the short run, and result in estimates that (correctly) suggest adequate financial protection in the current period, eventually these expenditures will have to be financed. The replenishment of assets and the repayment of loans may impose substantial financial hardship in subsequent periods.

Lastly, threshold levels used in here are based on the existing literature that does not mean that there is a kind of external validity of these thresholds. It will allow comparison (across location or time trends). However, there is no method as well that has been developed to assess the external validity of these thresholds. As a way forward, future research can concentrate firstly; on decomposing the causes of catastrophic expenditure secondly they can also look at social economic inequality in the determinants for catastrophic health care expenditure.

## Footnotes

1 For a full discussion visit; http://www.who.int/mediacentre/factsheets/fs395/en/

2 We used 2011 poverty line
